# Monitoring Dietary Intake and Physical Activity Electronically: Feasibility, Usability, and Ecological Validity of a Mobile-Based Ecological Momentary Assessment Tool

**DOI:** 10.2196/jmir.2617

**Published:** 2013-09-24

**Authors:** Jorinde Eline Spook, Theo Paulussen, Gerjo Kok, Pepijn Van Empelen

**Affiliations:** ^1^Faculty of Psychology and NeuroscienceDepartment of Work and Social PsychologyMaastricht UniversityMaastrichtNetherlands; ^2^TNOExpertise Group Life StyleLeidenNetherlands

**Keywords:** mobile-based Ecological Momentary Assessment (mEMA), feasibility, usability, ecological validity, dietary intake, physical activity

## Abstract

**Background:**

Despite the growing body of research on complex lifestyle behaviors (eg, Dietary Intake [DI] and Physical Activity [PA]), monitoring of these behaviors has been hampered by a lack of suitable methods. A possible solution to this deficiency is mobile-based Ecological Momentary Assessment (mEMA), which enables researchers to collect data on participants’ states in real-time by means of a smartphone application. However, feasibility, usability, and ecological validity need to be anticipated and managed in order to enhance the validity of mEMA.

**Objective:**

To examine the feasibility, usability, and ecological validity of a mEMA application (app) with regard to DI and PA among Dutch vocational education students.

**Methods:**

The students (n=30) participated in the mEMA study for seven consecutive days. They downloaded the mEMA app on their smartphone. Feasibility and usability of the mEMA app were evaluated by completing an online evaluation after seven days of participation. Ecological validity was measured by assessing the degree to which the content of the mEMA app approximated the real-world setting that was being examined, through several multiple-choice questions.

**Results:**

Compliance rates, as registered by the mEMA app, declined 46% over a seven-day period, while self-reported compliance, as measured with an online evaluation questionnaire afterwards, indicated a smaller decrease in compliance (29%). The students evaluated the mEMA app as feasible and usable. Ecological validity analyses showed that all DI and almost all PA multiple-choice options were covered with the compound response categories.

**Conclusions:**

The mEMA app offers the opportunity to assess complex health behaviors (eg, DI and PA) in real-time settings, in which specifically routinized behaviors are involved. However, the mEMA app faced several challenges that needed to be overcome in order to improve its validity. Overall, the present study showed that the mEMA app is a usable and ecologically valid tool to measure DI and PA behaviors among vocational education students, but compliance is still limited.

## Introduction

Self-report of lifestyle behaviors, affect, and cognitions is often biased (eg, recall bias, availability, and recency) [1-5]. For instance, studies of fruit and vegetable consumption and their underlying determinants have shown that people generally overestimate their own consumption and, as such, behavior is less well predicted among those with an optimistic bias [6]. Similar findings have been observed for fat consumption and physical activity [7,8]. Similarly, Nordgren et al (2008) showed in a study targeting smoking behavior that health cognitions are unstable and dynamic [9].

Hence, it is important to find ways to more reliably assess determinants and behavior, in order to better understand and change behaviors. A method that could reduce the threat of recall bias, availability, and recency effects is ambulatory monitoring, such as Ecological Momentary Assessment (EMA) [10]. As Stone, Shiffman, Atienza, and Nebeling state: “The core rationale for EMA methods rests on three core benefits of the EMA approach: (1) avoidance of recall and its attendant bias, by collecting data on momentary states; (2) realization of ecological validity by collecting data in the real world; and (3) achievement of temporal resolution, enabling analysis of dynamic processes over time” (page 6, [5]). As such, EMA enables assessment of complex health behaviors and related factors of influence in real-time settings [11] and appears to be applicable to often routinized behaviors [1]. In general, a comparison of EMA with traditional recall-based methodologies has shown that EMA produces more reliable results [3].

With the increasing popularity of smartphones, recent studies have indicated the usefulness of mobile-based Ecological Momentary Assessment (mEMA) [12-19]. The advantage of mEMA is that it is incorporated in a tool that is frequently used in daily living. However, researchers have also suggested that mEMA faces challenges that need to be anticipated and managed in order to enhance its validity (eg, time of day, day of the week, concurrent activities or states, nonresponse, and missing diary entries [5]). As such, it is essential to achieve adequate compliance and to study the feasibility, usability, and ecological validity of mEMA and mEMA measurements. Within this study, feasibility and usability refer to the practical application of mEMA in daily life (eg, monitoring burden). Ecological validity refers to the extent to which the data are representative of the possible range of experiences in daily life [5] and we explicitly focused on the extent to which we included the range of most relevant social and physical environments.

To date, use of mEMA in health promotion research is limited, especially when focusing on weight-related factors (ie, Dietary Intake [DI] and Physical Activity [PA]). To ensure that mEMA can be used effectively to monitor determinants of DI and PA in future research, it is imperative that these challenges are examined prior to its actual deployment [20]. To examine mEMA, the present study examined three research questions concerning the validity of a mEMA app: Is the mEMA app (1) a feasible (ie, what is the level of students’ compliance), (2) usable (ie, how is mEMA evaluated by the students), and (3) ecologically valid (ie, do the social and physical response items capture the most important day-to-day social and physical characteristics [5]) tool to measure determinants of DI and PA among vocational education students.

## Methods

### Participants

Data were collected from three vocational education schools in the Netherlands. Out of the 44 students who were approached to participate in the mEMA study, 30/44 students (68%) participated for 7 consecutive days (17/30, 57% were female), of which 17 students (aged 16-21 years) completed the online evaluation (regarding feasibility and usability of the mEMA app) after their 7 days of participation. Participatory incentives of ten €20 coupons were randomly distributed to the students at the end of the study. Those who participated for 7 days and filled in the online evaluation form doubled their chance of winning. All students consented to take part in the study and students aged ≤17 were provided with a passive consent form for their parents to complete. All procedures were approved by The Ethical Committee of Psychology (Maastricht University).

### Measures and Procedures

In a study conducted prior to the mEMA study (n=305 vocational education students), an assessment was made of the type of smartphone platforms that were most commonly used among the vocational education students. The majority of the students (244/305, 79.9%) indicated that they used a smartphone operating on BlackBerry OS, Android, or iOS (respectively 151/305, 49.5%, 55/305, 18.1%, and 37/305, 12.1%). Estimating an increased use of the iOS and Android platforms, the mEMA app was built on all three platforms.

### Mobile Ecological Momentary Assessment (mEMA) Study

**Figure 1:**
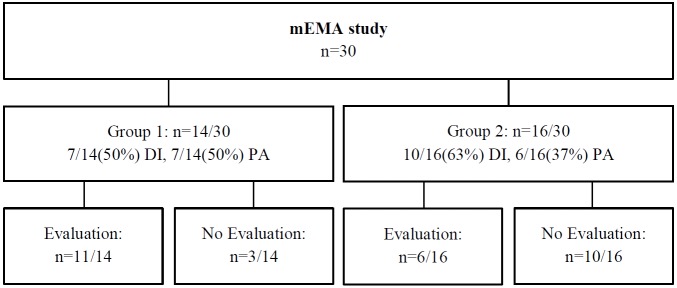
Study design and participant distribution.

In total, 44 students from three different vocational education schools in the Netherlands were approached by their teachers to participate in the present study, of which 30 students participated in the mEMA study. Students from the first two schools were allocated to the first group of participants (n=14) and were randomly assigned to the DI or PA condition (study design and participant distribution are illustrated in Figure 1). The students from the third school were allocated to the second group (n=16) and started using the mEMA app after several adjustments were made based on feedback from the first group (discussed in the Results section). Participants in the second group were encouraged to use the mEMA app similarly to those participating in the first group.

### Mobile Ecological Momentary Assessment App Use

Prior to the use of the mEMA app, students completed an online questionnaire regarding their DI or PA during the preceding 7 days. Data from this online questionnaire were collected for other study purposes. After completion, students who possessed a smartphone that operated on iOS (iPhone version 5.0), BlackBerry OS (version 6.0 or 7.0), or Android (version 2.2 and over) systems were eligible for participation, and downloaded the mEMA app from the BlackBerry OS, iOS, or Android app store (n=30). Students who possessed a smartphone with other operating systems or who did not own a smartphone were exempted from further participation. The students were invited to use the mEMA app for 7 consecutive days and gave their consent for participation. During these 7 days, participants were asked to fill in the same short questionnaires regarding their DI or PA during the preceding 3.5 hours, 5 times a day. Participants were able to close the app at any time.

### Evaluation

All students who participated in the mEMA study were asked to fill in an online evaluation form concerning the feasibility and usability (ie, functionality and interface) of the mEMA app. Additionally, after participants from the first group filled in the online evaluation form, group discussions took place per school, led by one of the researchers.

### Content Management System (CMS)

In order to upload content for the online questionnaire, mEMA app (both DI and PA conditions), and the evaluation, a content management system (CMS) was built. This CMS enabled researchers to monitor multiple complex health behaviors simultaneously (eg, DI and PA) and to adapt the content, text, and prompting sequence.

### Implementation Procedure for Using the mEMA App

An interval-contingent schedule was used for the mEMA app, which initially prompted participants at 5 time periods per day (8:00am, 12:00pm, 3:30pm, 6:30pm, and 9:30pm with a range of 30 minutes each), tailored to the schedule of their schools. These prompts were formatted as auditory signals and were displayed on their smartphone screens. When a diary entry was missed or rejected, up to two reminder signals were sent, once after 30 minutes and the second after 60 minutes. After missing and/or rejecting the two reminders, the CMS noted the initial diary entry as “missed”.

First, participants received instructions about the study and the use of the mEMA app (ie, how to download and use the app). They were instructed to start with an online questionnaire regarding their DI or PA during the preceding week. Second, students were able to use the app during the first day to get familiar with it. From the second day, the mEMA app started prompting participants, asking them to fill in a short questionnaire. Students were instructed to respond to these prompts as much as possible.

**Figure 2:**
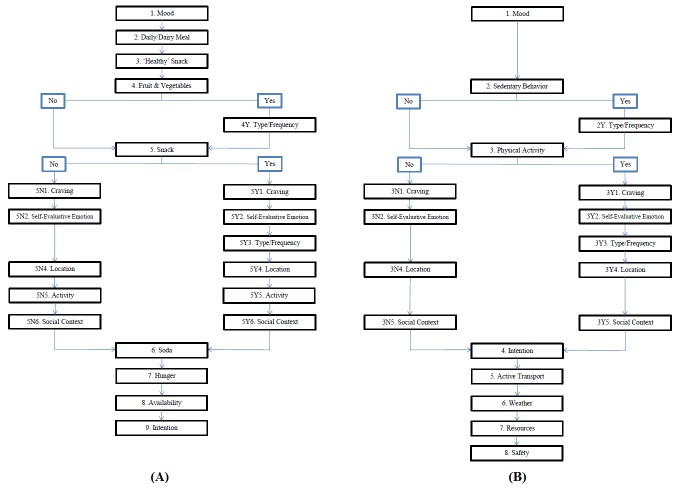
Decision trees of the mEMA app DI and PA questionnaire.

During each diary prompting sequence, students who were allocated to the DI condition received a total of 14-16 questions regarding their mood, eating behavior, food cravings, self-evaluative emotions, location, activities, social context, soft and energy drinks, snack intention, hunger, and the availability of food, respectively (ie, 12-14 questions depending on their responses within the decision tree; see Figure 2 A). These questions were derived from Carels et al, White et al, Dijkstra and Buunk, Adriaanse et al, Thomas, Thomas et al, and Rijpstra et al [21-26]. Students in the PA condition received 12-14 questions concerning their mood, sedentary behavior, physical activity, need for physical activity, evaluative emotions, location, social context, behavior intention, active transport, possible barriers, and feelings of security, respectively (see Figure 2 B). The PA questionnaires were derived from Carels et al, Cranford et al, De Vries et al, Dunton et al, Dunton et al, Grow et al, and Prins et al [21,27-32].

**Figure 3:**
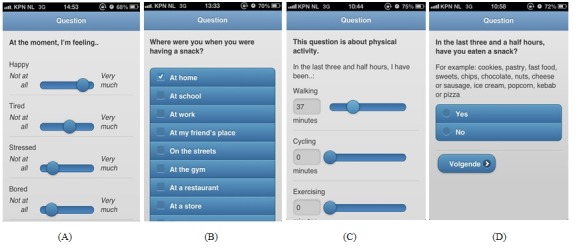
Screenshots of mEMA.

Four different types of response categories were used: Visual Analogue Scale (VAS slider) (see Figure 3 A), multiple-choice bullets (see Figure 3 B), multiple-choice open field combined with VAS slider (see Figure 3 C), and binary (see Figure 3 D). In the DI condition, 5-point (VAS slider) scales were used to measure mood, hunger, availability, and intention (eg, mood: “At the moment, I’m feeling happy”). Multiple-choice bullets were used as response categories for daily meal, self-evaluative emotions, location, activity, and social context (eg, location: “Where were you when you were having a snack?”). Multiple-choice open field combined with VAS sliders were used to indicate the type and frequency of fruits and vegetables, snacks, and sodas they had consumed (eg, “In the last three and a half hours, what type of snack(s) did you eat? And how many?”). These sliders ranged from 0-100, but the open fields enabled participants to fill in amounts that were larger than 100. Finally, binary response categories were used for decisions within the tree and food craving (eg, “In the last three and a half hours, have you eaten fruit or vegetables?”). All examples are translated from Dutch to English.

### Feasibility and Usability Measures

Feasibility and usability measures were derived from previously completed studies [15,16]. These measures contained 17 statements about the students’ subjective experience with the mEMA app (eg, “In my opinion, it was boring to work with the mEMA app”), the level of difficulty (eg, “In my opinion, the mEMA app was easy to use”), prompting sequence (eg, “In my opinion, the number of prompts that were sent during one week (5 times a day) was annoying”), length (eg, “In my opinion, the trial took too long”), and understanding (eg, “In my opinion, the questions were understandable”). Responses were scaled 1 (totally disagree) to 5 (totally agree). The midpoint of the scale was considered as a criteria for further interpretation (eg, scores above the midpoint of the usability scale were interpreted as “the mEMA app was easy to use”). Also, three open-ended questions were added to enable students to provide suggestions for further improvement of the mEMA app: (1) “Why did you ignore or postpone a prompt?”, (2) “I would make the following change to the mEMA app…”, and (3) “I would add the following to the mEMA app…”).

### Ecological Validity

Ecological validity was determined by assessing the degree to which the collected data represent the full-range of possible social and physical influences. The assessment for both DI and PA included two questions regarding the location and social context (eg, “Where were you while having a snack?” and “While you were being physically active, how many people were there with you?”). Response categories for both the location and social context were multiple choice and similar for DI and PA. Multiple-choice options included the following locations and social settings: at home, school, work, friend’s house, outside, restaurant, cafeteria, supermarket, sports club, or elsewhere; and a friend, colleague, classmate, team member, sibling, parent, partner, child, teacher, stranger, or other, respectively for the social setting.

### Data Analysis

SPSS version 20 was used for data analyses. Data from the mEMA app were transported from the smartphone into a secure computer system when participants had access to a Wi-Fi connection. Next, data from the computer system were converted to an SPSS database. With SPSS, descriptive statistics were generated (means and percentages) and compliance rates were calculated for the exploration of response patterns. Furthermore, a *t* test was done in order to examine gender differences in the perceived usability of the mEMA app. Prior to the analyses, normality was tested by means of Q-Q plots. Mean differences were analyzed using *t* tests when normally distributed and Mann-Whitney U test when they were non-normally distributed.

## Results

### Study Adjustments

After the first group had participated, technical problems were fixed and both feasibility and usability were evaluated by means of the aforementioned measurement procedures and group discussions. Based on the students’ feedback, two adjustments were made: (1) the number of daily prompts was reduced from 5 prompts a day to 4 prompts a day, and (2) the time sequence was reduced from 8 to 7 days of participation, as the start and evaluation of the study took place during their physical activity classes (once a week). Also, students evaluated the VAS sliders (see Figure 3 A, C) as bothersome. However, due to practical constraints (ie, Likert scales did not fit within the boundaries of all smartphone screens), usage of VAS sliders was maintained. The second group made use of the same mEMA app as the first group, but they were prompted 4 times a day during 7 consecutive days, instead of 5 prompts a day during 8 consecutive days.

An independent sample *t* test was conducted to test whether the usability of the mEMA app increased after the number of prompts was decreased from 5 to 4 times a day and the time sequence was reduced from 8 to 7 consecutive days. The results showed a significant difference between the first group (n=11) and the second group (n=6) regarding the app’s usability (*t*_14_=–2.15, *P=*.05). The second group perceived the app as easier to use (mean 4.33, SD 0.82) compared to the first group (mean 3.36, SD 0.92). However, no significant differences between the first group and the second group were found regarding the duration of the study (*P*=.19) and their evaluation (ie, how annoying) of the amount of prompting (*P*=.86).

### Compliance Rate

**Figure 4:**
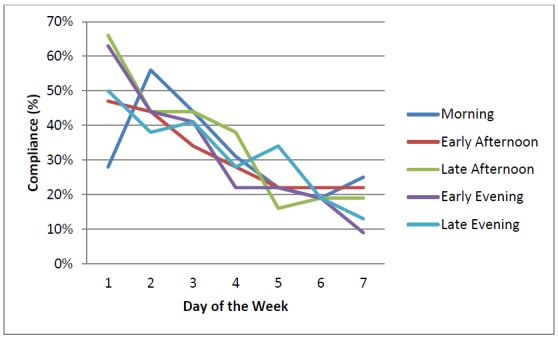
Response rates per times of day (n=30).

Students’ compliance was reported per day (n=30). Thirty participants started using the mEMA app at Day 1 (100%), and 7 days later, 14 students still participated (44%). Compliance decreased 56%. Further exploration of times of day did not indicate clear differences in compliance between morning, early afternoon, late afternoon, early evening, and late evening (56%, 55%, 61%, 55%, and 56% respectively). The response patterns per time of day over 7 days of participation are projected in Figure 4. Interestingly, the mEMA app was used 23 times without being prompted (ie, user based). The overall mean completion time per response was 138.7 seconds (SD 65.6; for DI [mean 132.3, SD 63.7] and PA [mean 146.8, SD 67.4]).

Total response rates were assessed by calculating the percentage of answered prompts per day. From Day 1 through 7, total response rates were 63%, 54%, 48%, 35%, 31%, 23%, and 23% respectively (n=128 prompts per day). Over time, the total response rate decreased 40%.

### Feasibility and Usability

Of all participants (n=30), 17 students (57%) completed the online evaluation form (ie, regarding the feasibility and usability of mEMA). Normality was assessed by means of Q-Q plots. These plots showed all items to be normally distributed. The mean scores (and standard deviations) of all items are displayed in Table 1. Participants reported that the mEMA app was relatively easy to use (mean 3.88, SD 0.93), that during the study they carried their smartphone with them every day (mean 4.65, SD 0.61), and that, according to them, the mEMA app worked well (mean 3.76, SD 1.09). Participants who took more time to complete the mEMA app questionnaires were also more likely to report that the time required to complete them was too lengthy (*r*=.154, *P*=.04). No significant differences in the perceived usability of the mEMA app between boys (mean 3.29, SD 0.95) and girls (mean 3.90, SD 9.99) were found (*P*=.88).

**Table 1:** Feasibility and usability measures (n=17).

Item	Mean score^a^(SD)	Score>mean^b^ *,* %
1. The mEMA app is easy to use.	3.88 (0.93)	65
2. It is easy to carry the smartphone with me.	4.35 (1.06)	65
3. I carried my smartphone with me every day.	4.65 (0.61)	71
4. After the researcher’s explanation I understood how the app would work.	4.24 (0.75)	41
5. It was fun to work with the app.	2.88 (1.27)	59
6. It was boring to work with the app.	2.88 (1.27)	59
7. The app worked well.	3.76 (1.09)	59
8. I experienced the prompts as reasonable.	3.00 (1.17)	71
9. The number of prompts was annoying.	3.47 (1.51)	53
10. I filled in the mEMA app questionnaires for 7 consecutive days.	3.76 (1.35)	71
11. I filled in the mEMA app questionnaires 4 times a day.	3.41 (1.42)	59
12. It was easy to fill in the mEMA app questionnaire on my smartphone.	4.24 (0.90)	47
13. The questions were well-displayed on my smartphone.	4.71 (0.47)	71
14. Filling in a mEMA app questionnaire was an interruption of my daily activities.	2.59 (1.46)	47
15. Filling in 1 mEMA app questionnaire took too long.	2.24 (1.20)	41
16. The study took too long.	2.24 (1.15)	35
17. I understood the questions that were asked.	4.71 (0.49)	77

^a^Scores were based on a 5-point Likert scale (ranging from 1 - totally disagree to 5 - totally agree).

^b^Score>mean illustrates the percentage of scores above the mean.

### Ecological Validity Analyses

Ecological validity analyses were performed to indicate whether response categories covered all types of students’ snacking in the DI condition and if response categories in the PA condition covered all types of physical activity. Table 2 shows that the consumed snacks could be categorized as cookies, pastries, fast food, sweets, chips, chocolate, nuts, cheese/sausage, popcorn, and kebab/pizza. Out of all prompts that participants responded to (128 out of 1020 prompts), 58 prompts were answered with “Yes, I have consumed an unhealthy snack during the past 3.5 hours”. In total, 104 snacks were reported by participants, indicating that per snacking episode an average number of 1.8 snacks were consumed.

The reported PA could be broken down into the categories of walking, exercising, working (standing), biking, cleaning the house, doing groceries, shopping, and something else (eg, internship at a kindergarten; see Table 2). Out of all prompts that participants responded to (n=128), 48 prompts were answered with “Yes, I have been physically active during the past 3.5 hours”. In total, 97 activities were reported, indicating that during several of these 48 time periods of 3.5 hours each, approximately 2 different activities were performed.

**Table 2:** Vocational education school students’ Dietary Intake (DI) and Physical Activity (PA).

Condition	Category	Frequency
**DI (n=58)**		
	Cookies	19
	Pastry	19
	Fast food	18
	Sweets	12
	Chips	11
	Chocolate	6
	Nuts	6
	Cheese/Sausage	4
	Ice cream	4
	Popcorn	3
	Kebab/Turkish Pizza	2
	Total DI	104
**PA (n=48)**		
	Walking	28
	Sporting	22
	Working (standing)	15
	Biking	13
	Cleaning the house	11
	Doing groceries	3
	Shopping	3
	Miscellaneous	2
	Total PA	97

### Social and Physical Categories

In addition to the ecological validity of response categories for DI and PA, ecological validity was analyzed for social context and location. These two context-related categories refer to the people that joined the participants and their exact location while they consumed a snack or were physically active. Results indicate that response categories for the social context fitted with 93% of all responses for DI and 98.3% for PA. Remaining responses were categorized as “Other” (including “family”, “people from school”, and “grandma” for DI, and “people from the gym” for PA). Regarding the location in which participants consumed snacks or were physically active, the developed response categories fitted with 90% for DI and 96% for PA. Remaining responses were categorized as “Elsewhere” (including “family”, “car”, “theater”, and “camping site” for DI, and “gym” and “in town” for PA).

Table 3 provides an overview of the categorized social contexts and locations. The overall amount of DI and PA by all participants appeared to be higher than the amount of reported social contexts and locations, indicating that participants might have eaten different snacks and have performed different types of PA in the same setting.

**Table 3:** Dietary Intake (DI) and Physical Activity (PA) related to social context and location.

Condition	Social Context^a^	n	Location	n
**DI**				
	Alone	9	Home	23
	Friend	9	School	7
	Colleague	11	Work	12
	Classmate	5	Friend’s	4
	Teammate	0	At the streets	5
	Sibling	8	Sports club	1
	Parent	14	Restaurant	2
	Partner	4	Mall	0
	Child	5	Elsewhere	6
	Teacher	0	-	-
	Stranger	0	-	-
**PA**				
	Alone	14	Home	18
	Friend	10	School	8
	Colleague	8	Work	11
	Classmate	5	Friend’s	8
	Teammate	4	At the streets	8
	Sibling	3	Sports club	14
	Parent	4	Restaurant	0
	Partner	8	Mall	0
	Child	0	Elsewhere	3
	Teacher	1	-	-
	Stranger	2	-	-

^a^Multiple answers are allowed per data collection point.

## Discussion

### Principal Findings

In line with technological developments over the past few years, the mEMA technique is gaining in popularity with claims of better accuracy in ambulatory data collection [7]. The mEMA approach offers the opportunity to assess complex health behaviors in real-time settings, in which specifically routinized behaviors are involved (eg, DI and PA) [1]. However, the mEMA app faced several challenges that needed to be overcome in order to improve its validity. The present study showed that the mEMA app is a usable and ecologically valid tool to measure DI and PA behaviors among vocational education students, but compliance is still limited.

Vocational education students’ self-rated compliance was 70.6% and is in accordance with compliance rates of previous electronic EMA studies [33]. However, the registered compliance rates of the in situ registration by the mEMA app indicated a compliance rate of only 43.8%. Such a discrepancy may be explained by an availability heuristic, indicating an overestimation of compliance when students evaluate their compliance retrospectively as with the online evaluation form used in this study. According to Stone et al (2007), noncompliance might be caused by several factors, eg forgetting and monitoring burden [5]. Up to 3 reminder signals were programmed in the mEMA app when participants did not respond, with time intervals of 30 minutes. Therefore, we assume that the chance of forgetting to respond to a mEMA prompt as a cause of noncompliance in the present study was small. Additionally, the number of prompts was reduced from 5 times a day to 4 times a day, based on feedback received from the first group. Accordingly, a significant decrease in monitoring burden was expected. However, the results from the online evaluation indicated that more than half of the students who filled in the online evaluation form (52.3%) still experienced the number of prompts as bothersome. Therefore, monitoring burden might be a cause of the students’ noncompliance in the present study. Noncompliance in the present study might also be attributable to the educational level of the students, as higher noncompliance rates have been more commonly reported among less educated students [34].

The usability of mEMA was rated as good. However, it should be mentioned that on the online evaluation form participants commented that the VAS-slider (see Figure 3 A, C) was “difficult and sometimes bothersome to use.” These comments are in line with experiences from adolescents who participated in an electronic chronic pain diary study [33]. These adolescents also perceived the VAS slider as “hard to control” and commented that when they dragged the slider, it did not move the way they wanted. However, they also mentioned that once they discovered how to use the VAS slider more effectively, they “got the hang of it” (page 300, [33]). Stinson et al suggested that VAS slider use could be improved by creating a thicker slider so it would move more easily, changing several sliders into radio buttons, and adding space for adolescents to enter their own responses. These adaptations were taken into account during the development of the mEMA app. But, despite these adaptations and rehearsal time prior to the study, the slider was still perceived as difficult and sometimes annoying to use. However, when the students received an explanation of the VAS sliders after they participated in the study (ie, due to practical limitations), support for the slider increased. Therefore, irritation might be prevented by providing a good rationale prior to study, including an explanation of ways to use the slider effectively. In addition to providing a good rationale, future studies are encouraged to develop new 5-point scale designs on smartphones, in order to find a viable alternative to VAS sliders.

To our knowledge, empirical research on the ecological validity of mEMA is still lacking. In the present study, the response categories used within the contexts of DI and PA appeared to cover (almost) all responses. This indicates that the collected data represents the full range of social and physical factors of influence. These categories could be used in future mEMA research on DI and PA behavior.

Besides the response categories used within the contexts of DI and PA, the mEMA app has potential use for future research on complex cognitions and (health-related) behaviors for behavioral research in general. The rationale for using mEMA rests on three central benefits of this methodology. First, retrospective recall (and the associated biases) is greatly reduced with mEMA because people report on current or recent states and events that occurred shortly prior to the received prompt. Second, mEMA occurs in natural settings, increasing external and ecological validity compared to clinical settings (eg, laboratory research). Third, multiple assessments occur over time, so temporal relationships among variables can be explored [3,35]. Fourth, the use of mobile-based EMA offers the opportunity to examine cognitions, affect, and behavior in the everyday context of people, using the natural handling and carrying of mobile phones. As such, mEMA offers a good opportunity for those behavioral, social, and health scientists and practitioners who aim to understand, intervene, and evaluate the effects of interventions on behavior (change), to examine individual differences as well as contextual differences.

### Limitations

Finally, important limitations of the present study need to be addressed. First, not all students were able to participate in the mEMA study, because not all students possessed a smartphone that operated on (recent versions of) BlackBerry, Android, or iOS. Consequently, 14 out of 44 students were exempted from further participation in the mEMA study. In order to include these exempted students in future research, a solution may be found in the (temporary) provision of eligible smartphones. Second, the mEMA app may reduce the likelihood of various biases (eg, recall bias, availability, and recency effects), but other biases may still threaten the validity of responses, such as social desirability. Social desirability bias might play an important role in health-related behavior as, for instance, participants are expected to underreport their dietary intake and overestimate their physical activity [6-8,36,37]. However, Crutzen et al (2010) indicated with their study on social desirability and self-reported health risk behaviors in Web-based research that there was no meaningful association between social desirability and self-reported health risk behaviors in Web-based research [34]. Because students in the present study used the mEMA app in real time in the absence of the researcher, it might be that a meaningful association between social desirability and self-reported DI and PA in mEMA studies is lacking too. Therefore, a comparable study on social desirability in future mEMA research might be interesting. Nevertheless, correction for these types of biases might still be necessary. Third, time-based prompting and prompting frequency were adjusted to the school’s schedule. However, in the Dutch vocational education system, students are required to work as interns for 2 days a week, which, according to our participants, prevented them from responding to all prompting sequences. Some students also mentioned their spring break as a reason for low compliance. Accordingly, flexible prompting (ie, adjustable by the participant) or encouragement of user-based entries could be taken into account in future deployments.

### Conclusion

Overall, mEMA offers the opportunity to assess complex health behaviors (eg, DI and PA) in real-time settings, in which specifically routinized behaviors are involved. However, the study also revealed some challenges with regard to use of the mEMA app that need to be taken into account in order to improve its validity. In particular, compliance is a reason for concern.

## Abbreviations

CMS: content management system
DI: dietary intake
EMA: Ecological Momentary Assessment
mEMA: mobile Ecological Momentary Assessment
PA: physical activity
VAS: visual analogue scale

## References

[1] Stone AA, Shiffman S (2002). Capturing momentary, self-report data: a proposal for reporting guidelines. Ann Behav Med.

[2] Bogers RP, Brug J, van Assema P, Dagnelie PC (2004). Explaining fruit and vegetable consumption: the theory of planned behaviour and misconception of personal intake levels. Appetite.

[3] Shiffman S, Stone AA, Hufford MR (2008). Ecological momentary assessment. Annu Rev Clin Psychol.

[4] Haedt-Matt AA, Keel PK (2011). Revisiting the affect regulation model of binge eating: a meta-analysis of studies using ecological momentary assessment. Psychol Bull.

[5] Stone AA, Shiffman S, Atienza AA, Nebeling L (2007). The science of real-time data capture: self-reports in health research.

[6] Lechner L, Brug J, De Vries H (1997). Misconceptions of fruit and vegetable consumption: differences between objective and subjective estimation of intake. Journal of Nutrition Education.

[7] Brug J, Van Assema P, Frewer LJ, Risvik E, Schifferstein H (2001). Beliefs about fat. Why do we hold beliefs about fat and why and how do we study these beliefs?. Food, People & Society.

[8] Ronda G, Van Assema P, Brug J (2001). Stages of change, psychological factors and awareness of physical activity levels in the Netherlands. Health Promotion International.

[9] Nordgren LF, van der Pligt J, van Harreveld F (2008). The instability of health cognitions: visceral states influence self-efficacy and related health beliefs. Health Psychol.

[10] Stone AA, Shiffman S (1994). Ecological momentary assessment in behavioral medicine. Annals of Behavioral Medicine.

[11] Serre F, Fatseas M, Debrabant R, Alexandre JM, Auriacombe M, Swendsen J (2012). Ecological momentary assessment in alcohol, tobacco, cannabis and opiate dependence: a comparison of feasibility and validity. Drug Alcohol Depend.

[12] Collins RL, Kashdan TB, Gollnisch G (2003). The feasibility of using cellular phones to collect ecological momentary assessment data: application to alcohol consumption. Exp Clin Psychopharmacol.

[13] Rofey DL, Hull EE, Phillips J, Vogt K, Silk JS, Dahl RE (2010). Utilizing Ecological Momentary Assessment in pediatric obesity to quantify behavior, emotion, and sleep. Obesity (Silver Spring).

[14] Daugherty BL, Schap TE, Ettienne-Gittens R, Zhu FM, Bosch M, Delp EJ, Ebert DS, Kerr DA, Boushey CJ (2012). Novel technologies for assessing dietary intake: evaluating the usability of a mobile telephone food record among adults and adolescents. J Med Internet Res.

[15] Heinonen R, Luoto R, Lindfors P, Nygård CH (2012). Usability and feasibility of mobile phone diaries in an experimental physical exercise study. Telemed J E Health.

[16] Tsai CC, Lee G, Raab F, Norman GJ, Sohn T, Griswold WG, Patrick K (2007). Usability and Feasibility of PmEB: A Mobile Phone Application for Monitoring Real Time Caloric Balance. Mobile Netw Appl.

[17] Zhang S, Wu Q, van Velthoven MH, Chen L, Car J, Rudan I, Zhang Y, Li Y, Scherpbier RW (2012). Smartphone versus pen-and-paper data collection of infant feeding practices in rural China. J Med Internet Res.

[18] Ozdalga E, Ozdalga A, Ahuja N (2012). The smartphone in medicine: a review of current and potential use among physicians and students. J Med Internet Res.

[19] Schnall R, Okoniewski A, Tiase V, Low A, Rodriguez M, Kaplan S (2013). Using text messaging to assess adolescents' health information needs: an ecological momentary assessment. J Med Internet Res.

[20] Mays D, Cremeens J, Usdan S, Martin RJ, Arriola KJ, Bernhardt JM (2010). The feasibility of assessing alcohol use among college students using wireless mobile devices: Implications for health education and behavioural research. Health Education Journal.

[21] Carels RA, Hoffman J, Collins A, Raber AC, Cacciapaglia H, O'Brien WH (2001). Ecological momentary assessment of temptation and lapse in dieting. Eat Behav.

[22] White MA, Whisenhunt BL, Williamson DA, Greenway FL, Netemeyer RG (2002). Development and validation of the food-craving inventory. Obes Res.

[23] Dijkstra A, Buunk AP (2008). Self-evaluative emotions and expectations about self-evaluative emotions in health-behaviour change. Br J Soc Psychol.

[24] Adriaanse MA, de Ridder DT, de Wit JB (2009). Finding the critical cue: implementation intentions to change one's diet work best when tailored to personally relevant reasons for unhealthy eating. Pers Soc Psychol Bull.

[25] Thomas JG (2009). Toward a better understanding of the development of overweight: a study of eating behavior in the natural environment using ecological momentary assessment.

[26] Thomas JG, Doshi S, Crosby RD, Lowe MR (2011). Ecological momentary assessment of obesogenic eating behavior: combining person-specific and environmental predictors. Obesity (Silver Spring).

[27] Cranford JA, Shrout PE, Iida M, Rafaeli E, Yip T, Bolger N (2006). A procedure for evaluating sensitivity to within-person change: can mood measures in diary studies detect change reliably?. Pers Soc Psychol Bull.

[28] de Vries SI, Bakker I, van Mechelen W, Hopman-Rock M (2007). Determinants of activity-friendly neighborhoods for children: results from the SPACE study. Am J Health Promot.

[29] Dunton GF, Whalen CK, Jamner LD, Floro JN (2007). Mapping the social and physical contexts of physical activity across adolescence using ecological momentary assessment. Ann Behav Med.

[30] Grow HM, Saelens BE, Kerr J, Durant NH, Norman GJ, Sallis JF (2008). Where are youth active? Roles of proximity, active transport, and built environment. Med Sci Sports Exerc.

[31] Prins RG, van Empelen P, Beenackers MA, Brug J, Oenema A (2010). Systematic Development of the YouRAction program, a computer-tailored physical activity promotion intervention for Dutch adolescents, targeting personal motivations and environmental opportunities. BMC Public Health.

[32] Dunton GF, Liao Y, Intille SS, Spruijt-Metz D, Pentz M (2011). Investigating children's physical activity and sedentary behavior using ecological momentary assessment with mobile phones. Obesity (Silver Spring).

[33] Stinson JN, Petroz GC, Tait G, Feldman BM, Streiner D, McGrath PJ, Stevens BJ (2006). e-Ouch: usability testing of an electronic chronic pain diary for adolescents with arthritis. Clin J Pain.

[34] Crutzen R, Göritz AS (2010). Social desirability and self-reported health risk behaviors in web-based research: three longitudinal studies. BMC Public Health.

[35] Heron KE, Smyth JM (2010). Ecological momentary interventions: incorporating mobile technology into psychosocial and health behaviour treatments. Br J Health Psychol.

[36] Tooze JA, Subar AF, Thompson FE, Troiano R, Schatzkin A, Kipnis V (2004). Psychosocial predictors of energy underreporting in a large doubly labeled water study. Am J Clin Nutr.

[37] Sallis JF, Saelens BE (2000). Assessment of physical activity by self-report: status, limitations, and future directions. Res Q Exerc Sport.

